# A tetravalent nanoparticle vaccine elicits a balanced and potent immune response against dengue viruses without inducing antibody-dependent enhancement

**DOI:** 10.3389/fimmu.2023.1193175

**Published:** 2023-05-19

**Authors:** Qier Chen, Rong Li, Bolin Wu, Xu Zhang, Hui Zhang, Ran Chen

**Affiliations:** ^1^ Institute of Human Virology, Department of Pathogen Biology and Biosecurity, Key Laboratory of Tropical Disease Control of Ministry Education, Guangdong Engineering Research Center for Antimicrobial Agent and Immunotechnology, Zhongshan School of Medicine, Sun Yat-sen University, Guangzhou, Guangdong, China; ^2^ Guangzhou National Laboratory, Bio-Island, Guangzhou, Guangdong, China

**Keywords:** DENV, nanoparticle vaccine, ferritin, ADE, adjuvant

## Abstract

Dengue fever is a global health threat caused by the dengue virus (DENV), a vector-borne and single-stranded RNA virus. Development of a safe and efficacious vaccine against DENV is a demanding challenge. The greatest pitfall in the development of vaccines is antibody-dependent enhancement (ADE), which is closely associated with disease exacerbation. We displayed the modified envelope proteins from the four serotypes of the DENV on a 24-mer ferritin nanoparticle, respectively. This tetravalent nanoparticle vaccine induced potent humoral and cellular immunity in mice without ADE and conferred efficient protection against the lethal challenge of DENV-2 and DENV-3 in AG6 mice. Further exploration of immunization strategies showed that even single-dose vaccination could reduce pathologic damage in BALB/c mice infected with high doses of DENV-2. Treatment with cyclic-di-guanosine monophosphate facilitated a higher titer of neutralizing antibodies and a stronger type-1 T-helper cell-biased immune response, thereby revealing it to be an effective adjuvant for dengue nanoparticle vaccines. These data suggest that a promising tetravalent nanoparticle vaccine could be produced to prevent DENV infection.

## 1 Introduction

The dengue virus (DENV) belongs to the family *Flaviviridae* and genus *Flavivirus* and has four serotypes (DENV 1–4). Dengue fever (DF) is an acute mosquito-borne viral disease caused by DENV, which is transmitted predominantly by *Aedes aegypti* and *Aedes albopictus*. Most DENV infections are asymptomatic or result in self-limited diseases but may also develop into serious and potentially lethal situations, such as dengue hemorrhagic fever or dengue shock syndrome (1, 2).

According to one recent estimate, ~390 million people are at risk of DENV infection (3). The wide spread of the DENV poses a threat to human health and the global economy. Approved antiviral therapies for DENV infection are lacking. Development of a DENV vaccine has been challenged by the need to generate a balanced protective immunity against the four distinct DENV serotypes (2, 4). In recent decades, many candidates for dengue vaccines have been reported: live attenuated viruses (LAVs), purified inactivated viruses (PIVs), nucleic-acid vaccines, and vaccines based on the subunits of nucleic acids.

A major drawback in the development of a DENV vaccine is antibody-dependent enhancement (ADE). ADE occurs if a secondary infection is followed by a heterotypic serotype. It can cause a higher risk of severe disease by enhancing the entry and replication of viruses. ADE is mediated by non-neutralizing antibodies generated after the primary DENV infection (5, 6). The LAV vaccine CYD-TDV (Dengvaxia™) was the first licensed dengue vaccine and was followed by two second-generation LAVs (TAK-003 and TV003/TV005). However, LAV replication is different among DENV strains and may result in unbalanced immunity against the other serotypes, which increases the risk of severe DF among the vaccinated dengue-naïve population due to ADE (6). Compared with LAVs, PIVs can avoid the interference of replication imbalance, but the low immunogenicity hinders their further development (7). Nucleic-acid vaccines can be DNA vaccines or mRNA vaccines. DENV DNA vaccines are poorly immunogenic, which was verified in two dengue DNA vaccines (TVDV, D1ME100) in a phase-I clinical trial (4, 8). A DENV mRNA vaccine based on non-structural regions has been shown to induce strong T-cell responses in human transgenic mice, but whether antibodies are associated with ADE is not known (9). With regard to vaccines based on nucleic-acid subunits, researchers have focused on the in DENV envelope protein (E protein), which consists of three domains (DI, DII, DIII). The DI domain contains some important residues, including N153 (glycosylated asparagine residue), K291 and K295, which are involved in interaction with the cell surface (10, 11). Often, DII induces sub-cross-antibodies to cause ADE, whereas the DIII domain is mainly responsible for receptor binding and is the target for specific neutralizing antibodies (nAbs) (12, 13).

Several DIII domain-based nanoparticle vaccines have been reported (14, 15). However, clinical studies have shown that the E protein DIII domain (EDIII)-targeted antibody accounts for only a small fraction of nAbs in convalescent patients infected naturally with the DENV (6, 16). Increasing numbers of studies have revealed that many complex epitopes in the E protein (except for the DIII domain) of the DENV are important to establish protective immune responses (6, 17, 18). All the problems described above must be solved if a DENV vaccine is to be developed.

In recent years, the ferritin-based nanoparticle platform has shown great advantages in the development of vaccines against severe acute respiratory syndrome-coronavirus-2 (SARS-CoV-2) as well as Zika, influenza, and Epstein–Barr viruses (19–22). Previously, we showed that ferritin and the receptor-binding domain (RBD) in the S protein of SARS-CoV-2 were expressed separately but could conjugate covalently based on a GvTagOpti/SdCatcher (Gv/Sd) system (20, 23, 24). The target antigens were displayed on the surface of 24-mer ferritin and elicited strong immunogenicity *in vivo*. We postulated that our ferritin-based nanoparticle platform could provide a promising strategy for development of a DENV vaccine. Therefore, we modified the E protein of the four serotypes of the DENV and displayed them on the ferritin nanoparticle to develop a tetravalent DENV (E13-HPF nanoparticle) vaccine.

In the present study, the designed tetravalent DENV vaccine showed a great preventive effect but also avoided the risk of ADE significantly even though antibodies from immunized sera were diluted to a low concentration. Exploration of different immune strategies revealed that different levels of immunization could protect mice from DENV-2 efficiently. As an adjuvant, cyclic di guanosine monophosphate (c-di-GMP) could enhance the type-1 T helper (Th1) cell-biased immune response and improve the protective ability of the nanoparticle vaccine. In brief, we developed a tetravalent DENV vaccine providing balanced and effective immune protection without ADE.

## 2 Materials and methods

### 2.1 Cells and viruses

CHO-S, HEK293T, HEK293F, BHK-21 cells and Vero cells were obtained from ATCC. The adherent cells were cultured in Dulbecco’s modified Eagle medium (DMEM; Sigma) supplemented with 10% fetal bovine serum (FBS). CHO-S cells were cultured in CHO S4 medium (Duoning Biology) and HEK293F cells in 293 medium (Union), with 8 mM glutamine at 37°C in 5% CO2.

Four strains of dengue viruses [DENV-1(D19044), DENV-2(16681), DENV-3 (D191267) and DENV-4(GZ14D4)] were kindly provided by Professor Yiping Li (Sun Yat-sen University). DENV-1 was propagated in BHK-21 cells; DENV-2, DENV-3 and DENV-4 were propagated in Vero cells. Virus titers were quantified as described before and expressed as focus-forming units FFU/ml (25).

### 2.2 Protein expression and purification

Based on our recent works regarding ferritin-based nanoparticle vaccines, a similar dengue vaccine was constructed (20, 23, 24). Briefly, four sequences of E protein (1-397aa) from different dengue viruses (Dengue virus 1 Thailand/AHF 82-80/1980, Dengue virus 2 Thailand/16681/84, Dengue virus 3 Philippines/H87/1956, Dengue virus 4 Philippines/H241/1956) were chosen. Amino acid sequences of DII (53-132 aa and 191-280 aa) in E protein were respectively replaced by sequences of ‘GGGGS’ and ‘S’, to form the new E13 protein (DENV-1: E113, DENV-2: E213, DENV-3: E313, DENV-4: E413). Then the N-termini were fused by the sequences of secretory signal peptide, while the C-termini were followed by the sequences of GvTagOpti from GvTagOpti (Gv)/SdCatcher(sd) connection system described previously (26). Four reconstructed sequences of E13-Gv (DENV-1: E113-Gv, DENV-2: E213-Gv, DENV-3: E313-Gv, DENV-4: E413-Gv) were codon-optimized for mammalian cells and cloned into the vector pLVX plasmid. HEK293T cells were co-transfected with the constructed pLVX plasmids, the psPAX2 (Addgene) plasmid and PLP/VSVG by utilizing polyethyleneimine (PEI, Sigma). The recombinant viruses in the supernatant were collected after 48 h and then infected CHO-S cells for another 48 h (**Figure S1C**). After expending cultivation of the infected cells, the supernatant was harvested by centrifugation at the day 7.

For the expression of Sd-ferritin, the Sd gene was first fused to the N-terminus of the ferritin (HPF) gene, and the DNA sequence of Sd-ferritin was cloned into the pET28a vector. The constructed plasmid was transformed into BL21 (Takara) prokaryotic expression bacteria. Sd-ferritin was expressed from *E. coli*.

Both E13-Gv and Sd-ferritin were purified via Size Exclusion Chromatography (GE Healthcare) on a Superose 6 column. The purified protein was quantified by a BCA protein assay kit and the western blotting was performed to confirm the purification of the protein. Four purified E13-Gv in separate were irreversibly covalently conjugated to the Sd-ferritin to generate the E13-HPF nanoparticle which was further purified by Size Exclusion Chromatography and confirmed by the Coomassie blue staining. The tetravalent DENV nanoparticle vaccine (E13-HPF vaccine) consisted of a 1:1:1:1 mix of four E13-HPF nanoparticles.

For ELISpot assay, N-terminus of DENV-2 DIII protein was fused with 6-his-tag, and then the protein was expressed in HEK293F cells and purified with a nickel column.

### 2.3 Transmission electron microscopy

Transmission electron microscopy (TEM) grids of HPF and E13-HPF vaccine were proceeded to negative-stain electron microscopy. Briefly, each sample (0.05 µg/µl) was applied to glow-discharged electron microscopy grids covered by a thin layer of continuous film and stained with 2% uranyl acetate. Then images were recorded at a magnification of 120,000 × on Talos L120C microscope (ThermoFisher) operating at an acceleration voltage of 120 kV. Scale bars represented 100 nm.

### 2.4 Mice experiment

AG6 mice were a gift from Professor Zhiwei Wu (Nanjing University) and six-week-old female BALB/c mice were purchased from Gempharmatech Co., Ltd. These mice were raised in SPF facilities at the Laboratory Animal Center of Sun Yat-sen University. AG6 mice and BALB/c mice were immunized subcutaneously (s.c.) with 10 μg E13-HPF vaccine and 10 μg HPF proteins as control. Unless noted, antigens were mixed with an equal volume of Alum (InvivoGen) adjuvant. Sera were collected and heat inactivated at 56°C for 30 min.

For immunized AG6 mice challenge, 6×10^5^ FFU of DENV-2 16881 strain or 10^5^ FFU of DENV-3 D191267 strain were injected intravenously (i.v.) into each mouse. Survival analyses of the challenged AG6 mice were recorded every 2 days, including survival rate and clinical scoring. In order to detect the viremia 3 days post infection (dpi), AG6 mice serum was collected to exact RNA through the viral RNA kit (Magen).

For immunization strategy research, each immunized BALB/c mouse in different groups was conducted an intravenous injection of DENV-2 16881 strain (10^7^ FFU). Then BALB/c mice were sacrificed 7 dpi. Livers were harvested and fixed in 4% paraformaldehyde buffer (Servicebio) for 48 h, followed by embedding with paraffin. Longitudinal sections were performed on these tissues and sections (3-4 mm) were stained with hematoxylin and eosin (H&E).

### 2.5 Enzyme linked immunosorbent assay

Four types of 5 μg/ml E13 proteins were respectively coated on high-binding 96-well plates (Greiner Bio-one), overnight at 4°C. Then the plates were blocked with 100 μl 5% non-fat milk/PBS at room temperature for 1 h. After washing with PBS, immunized animal serum was serially diluted and added into each well at 37°C for 1 h. After washing with PBS/T (containing 0.1% Tween-20) three times, 100 μl HRP-conjugated goat anti-mouse secondary antibody (Invitrogen) at a dilution of 1:4000 was added at 37°C for another 1 h. The plates were washed four times before 50 μl TMB solution (eBioscience) was added to each well. After 5 min quenching reaction by adding 50 μl stop solution (Solarbio), measure absorption at 450 nm. GraphPad Prism 8.0 software for non-linear regression was used to calculate endpoint titers.

### 2.6 Focus reduction neutralizing test

This method was described previously in many studies (27, 28). Briefly, Vero cells or BHK-21 cells were seeded at 4×10^4^ cells/well in 96-well plates and incubated overnight at 37°C in 5% CO_2_. Mice serum was diluted at 1:10, then serially diluted 5-fold down a 96-well plate in serum-free OPTI-MEM. After adding the equal volume of dengue virus stocks at 70 FFU/well, the virus/serum mixture was incubated for 1h at 37°C. The confluent Vero cells were washed by PBS before the virus/serum mixture was added. Following another 1 h incubation at 37°C, the mixture was removed, and the cells were overlaid with 200 µl of 1.6% CMC in DMEM supplemented with 2% FBS. The plates were incubated for 2 days at 37°C.

After the supernatant was removed, the cells were fixed with 50 µl ice-cold methanol for 30 min at RT. After washing with 200 µl PBS, cells were incubated with 50 µl of rabbit anti-NS3 polyclonal antibody (GeneTex) which was 1:3000 diluted with PBS containing 1% BSA at 4°C overnight. Following three washes with PBS/T (0.1% Tween-20), 1:2000 diluted in PBS containing 1% BSA, FITC-conjugated secondary antibody (Abcam) was added 50µl to per well and the plates incubated for 1 h at 37°C. After washing 4 times with PBS/T, the plates were allowed to dry and imaged with ImmunoSpot microanalyzer. The 50% inhibition of infection of cells was represented by the decrease of number of DENV-infected cells in the sample wells compared to virus control wells. Reduction rates of the serial dilution assay were analyzed by Graphpad Prism 8.0 using non-linear regression to measure the NT50 titer.

### 2.7 Antibody-dependent enhancement assay

Primary cells were isolated from bone marrow of BALBc/c mice as described (29). To generate bone marrow derived macrophage (BMDM), the isolated cells (1×10^6^/well) were cultured in a 24-well plate for 7 days in RPMI 1640 (10% FBS, penicillin, streptomycin) containing 20 ng/ml murine macrophage-stimulating factor (M-CSF, PeproTech). On day 8, 200 µl of mice serum was diluted at 1:25 in RPMI 1640 and were incubated with the equal volume of diluted virus stocks (MOI=0.1) for 1 h at 37°C in 5% CO_2_, the virus-only well as mock control. After removing the supernatant, BMDM were incubated with the serum/virus mixture for another 1 h at 37°C. 400 µl of the RPMI 1640 (4% inactivated FBS, penicillin, streptomycin) was added to each well, and then the plates were allowed to maintain for 24 h at 37°C in 5% CO_2_. On the next day, the cells were collected, and total RNA was extracted with a RNA extraction reagent kit (Magen). The RNA was stored in -20°C to be analyzed.

### 2.8 Quantitative real-time PCR

To obtain cDNA for qRT-RCR analysis, for RNA from all the samples, reverse transcription was carried out using HiScript II First-strand cDNA Synthesis Kit. RNA was quantified by SYBR Green real-time PCR (Universal SYBR RT-RCR Master Kit) with strain-specific primers (**Table S2**). For ADE assays, viral RNA levels were normalized by housekeeping gene β-actin levels (β-actin: forward primer: 5’- GGCTGTATTCCCCTCCATCG-3’, reverse primer: 5’- CCAGTTGGTAACAATGCCATGT-3’). For viremia detection, the 3’-end specific gene of DENV-2(16881) or DENV-3(D191267) was cloned into a pMD-18T vector for standards. Then 10-fold serially diluted standards were subjected to SYBR Green real-time PCR to generate a standard curve for the quantification of viral RNA represented by the copy number. All the reactions were carried out on a QuantStudio 7 Flex System (Applied Biosystems) under the following reaction conditions: 95°C for 3 min, 40 cycles of 94°C for 15 s and 60°C for 30 s.

### 2.9 Flow cytometry

The spleen of each mouse was collected in PBS and homogenized, following incubation in ACKlysis buffer to remove red blood cells. Then splenocytes were stored in cell saving medium (KeyGEN) at -80°C until analysis. For analysis, cells were rapidly recovered at 37°C and balanced in RPMI 1640. After centrifugation, the cells were re-suspended with culture medium and remained at 37°C incubator for 0.5 h for complete cells recovery. Then the cells were washed with PBS and stained with the diluted fluorochrome-conjugated monoclonal antibodies for 30 min within PBS containing 0.5% BSA on ice. The following antibodies were used: eBioscience Fixable Viability Dye eFluor 780, anti-mouse CD3-AF647 (Biolegend), anti-mouse CD4-R718 (BD Biosciences), anti-mouse CD8-BV510 (Biolegend), anti-mouse CD62L-FITC (eBioscience), anti-mouse CD44-Percp/Cy5.5 (BD Biosciences), anti-mouse CD19-BV510 (Biolegend), anti-mouse B220-Percp/Cy5.5 (Biolegend) and anti-mouse CD38-R718 (BD Biosciences), anti-mouse IgG1-FITC (Biolegend) and anti-mouse IgG2a-AF647 (Biolegend). All flow cytometry data were acquired on FACS Aria II flow cytometer (BD Biosciences) and analyzed with FlowJo software.

### 2.10 Enzyme-linked immune absorbent spot assay

The splenocytes from each group (n=5) was collected at week-26. The splenocytes were cultured with DENV-2 DIII at a concentration of 10 µg/ml and co-stimulated with 2 mg/mL anti-CD28 (Biolegend) at 37°C with 5% CO_2_ for 20-24 h. Antigen specific splenocytes of BALB/c mice were detected by mouse IFN-γ ELISPOT kit and mouse IL-4 ELISPOT kit (Dayou) according to the manufacturer’s protocol. Antigen-specific spots were counted using ImmunoSpot microanalyzer. The number of spots was converted into the number of spots per 10^5^ cells.

### 2.11 Statistical analysis

Experiments were conducted independently in triplicate. Statistical analyses were conducted utilizing Graphpad Prism 8.0. The statistical details of specific experiments had been described in the figure legends and methods. All data are shown as the mean ± SEM. P values <0.05 were considered significant. ns: p>0.05, *p < 0.05, **p < 0.01, ***p < 0.001, ****p < 0.0001.

### 2.12 Ethics statement

The animal experiments were approved by the Ethics Committee of Zhongshan School of Medicine (ZSSOM) of Sun Yat-sen University on Laboratory Animal Care (Assurance Number: 2021002109).

## 3 Results

### 3.1 Construction and characterization of a tetravalent DENV nanoparticle vaccine

To construct a DENV nanoparticle vaccine, we modified the E protein of the DENV and displayed it on a ferritin-based nanoparticle. E protein is the main target of nAbs to block DENV infection. The DII domain of E protein is the conservative domain that induces antibodies to mediate ADE.

**Figure 1:**
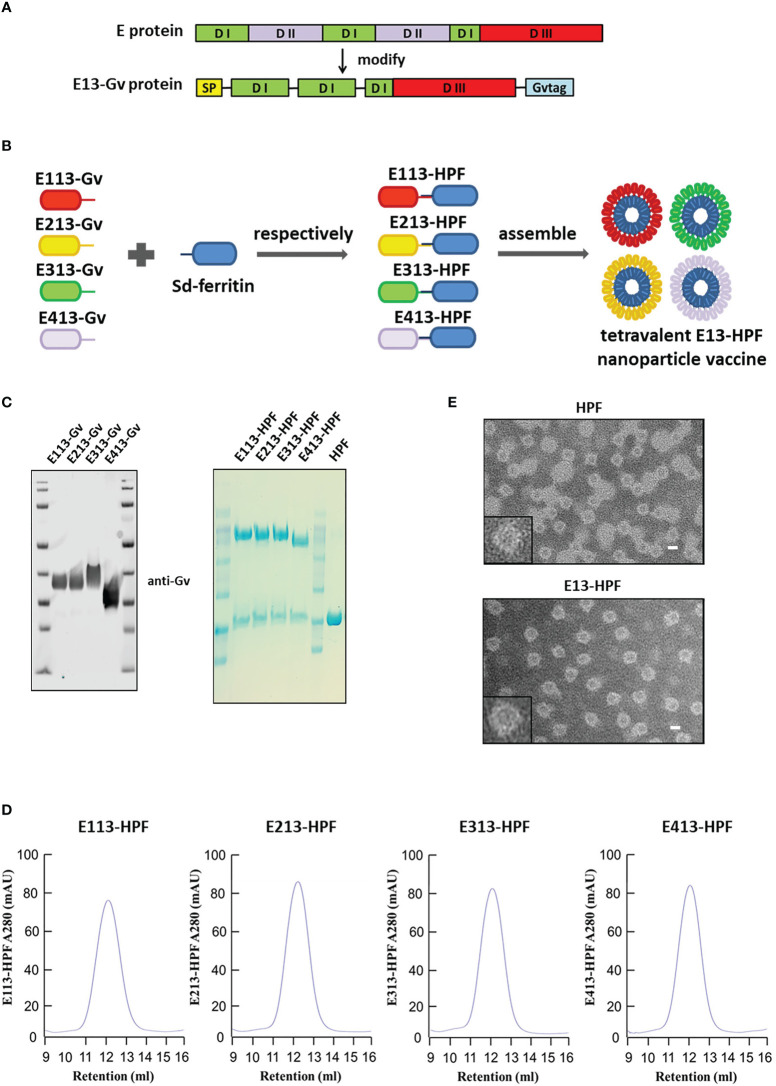
Construction and characterization of E13-HPF vaccine. **(A)** Design of E13-Gv protein. E protein consists of domain I (DI), domain I (DII), and domain III (DIII). In our design, DII was respectively replaced by the linker ‘GGGGS’ and amino acid ‘S’ and fused with a Gv sequence to construct the E13-Gv protein. SP, secretory signal peptide; Gvtag, GvTagOpti (Gv). **(B)** Nanoparticle vaccine (schematic). Four E13-Gv proteins were incubated with Sd-ferritin at 25°C overnight to form E13-HPF nanoparticles. Sd, SdCatcher; HPF, ferritin. DENV-1 (GenBank: BAA00394.1), DENV-2 (UniProtKB/Swiss-Prot: P29990.1), DENV-3 (UniProtKB/Swiss-Prot: P27915.1), DENV-4 (GenBank: AAX48017.1). **(C)** Western blotting of four E13-Gv proteins (left) and Coomassie Blue staining of four E13-HPF nanoparticles (right). **(D)** SEC of four E13-HPF nanoparticles. Ultraviolet absorption at 280 is shown and the retention volume represents the peak of each nanoparticle. **(E)** TEM images of HPF nanoparticles and E13-HPF vaccine. Scale bars represented 100 nm.

We replaced the DII domain with a linker in the full-length E protein to ensure the natural structure of the reconstructed E protein (E13 protein) (**Figures 1A**, **S1A**). The Sd-coding sequence was fused genetically to the N-termini of ferritin (HPF) (**Figure S1B**). The Gv-coding sequence was fused to the C-termini of the E13 sequences of DENV 1–4 (E113-Gv, E213-Gv, E313-Gv, E413-Gv) (**Figure 1B**). Four stable CHO-S cells, expressing the four serotypes of DENV E13-Gv, respectively, were constructed. The purity of proteins in the cell supernatant was high (**Figure S1C**). Size-exclusion chromatography (SEC) was conducted to purify all of these proteins. Protein expression was analyzed in western blotting using a homemade anti-Gv antibody (**Figure 1C**). The purified DENV 1–4 E13-Gv was irreversibly and covalently conjugated to Sd-ferritin to generate DENV 1–4 E13-HPF (E113-HPF, E213-HPF, E313-HPF, E413-HPF), followed by purification by SEC (**Figure 1D**). Due to covalent conjugation between GvTagOpti and SdCatcher [this system had been described in detail in our work (26)], E13-Gv was incubated with Sd-ferritin to form E13-HPF, which could self-assemble into a 24-mer nanoparticle (**Figure 1B**). The molecular weight of E13-HPF was ~70 kDa, whereas that of E13-Gv and Sd-ferritin was ~40 kDa and ~30 kDa, respectively (**Figure 1C**). Besides, the yield of E13-Gv was ~30 mg/L as measured with a BCA protein assay kit. SEC revealed a single and smooth peak but also showed the high purity of the four types of nanoparticles (**Figure 1D**). To generate the tetravalent DENV nanoparticle vaccine (E13-HPF vaccine), four E13-HPF nanoparticles were mixed in equimolar proportions. The morphology and homogeneity of E13-HPF vaccine were also verified by transmission electron microscopy (TEM) (**Figure 1E**).

### 3.2 E13-HPF vaccine induces potent humoral immune responses without causing ADE

**Figure 2:**
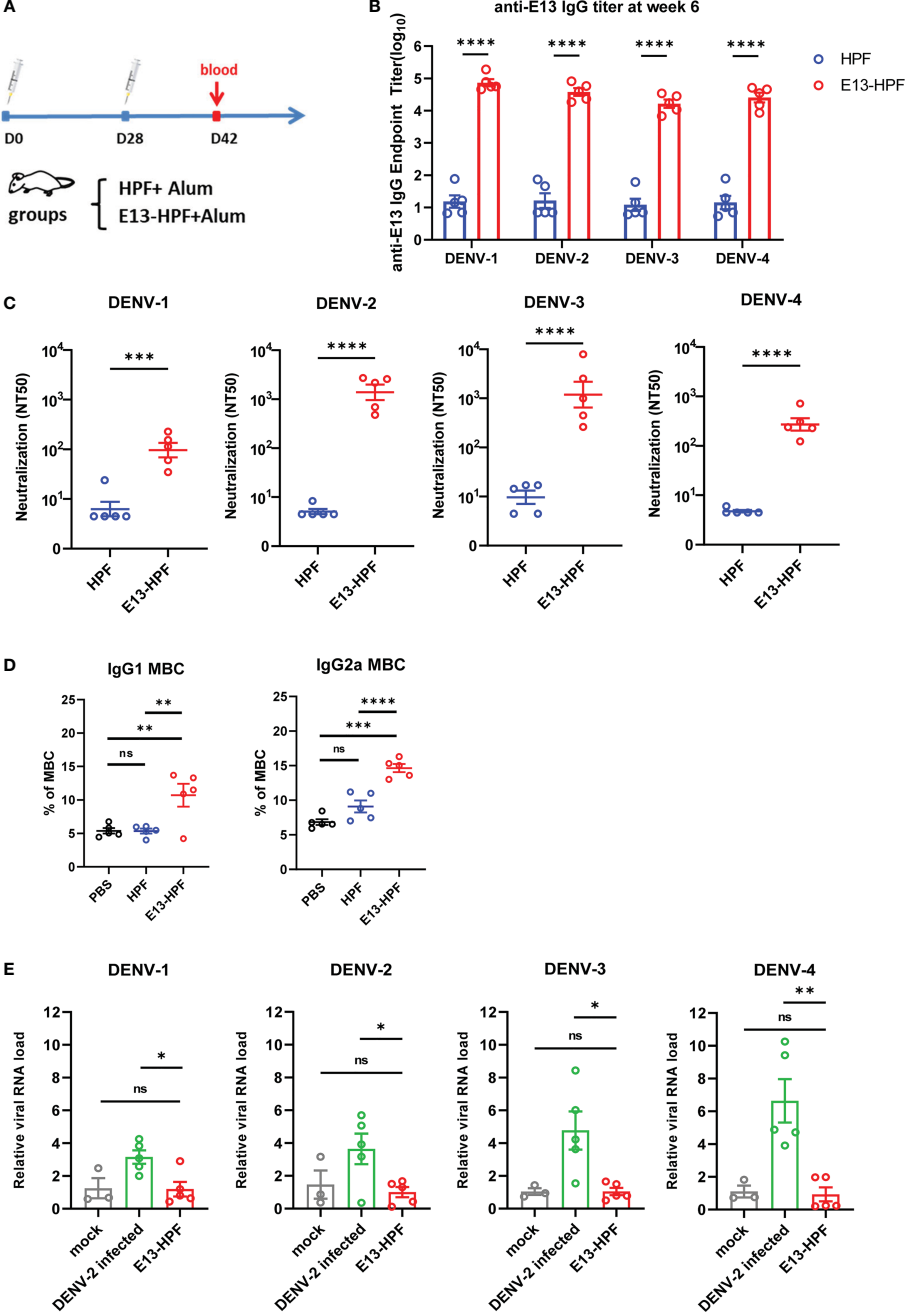
E13-HPF vaccine immunization induces potent humoral immune responses in BALB/c mice without ADE. **(A)** Vaccination schedule for BALB/c mice (schematic). Five mice in each group were immunized with 10 µg of tetravalent E13-HPF nanoparticle vaccine or HPF on day-0 and day-28. Sera were collected every 2 weeks. **(B)** ELISA detection of the anti-E13 IgG titer at week-6 (on day-42). Data are represented as the reciprocal of the endpoint serum dilution. Adjusted p-values were calculated by two-way ANOVA with Sidak’s multiple-comparisons test. **(C)** NT50 of neutralizing antibodies against four-serotype DENV in each group was determined by FNRT and represented as the half-maximal inhibitory concentration (IC_50_) on day-42. Data were analyzed by the Student’s t-test. **(D)** Percentages of total IgG1^+^ MBCs and IgG2a^+^ MBCs (CD19^+^ B220^+^ CD38^+^) within the spleen of mice in each vaccine group 2 weeks post-boost vaccination (n = 5). Mice immunization of PBS with alum was also as control. **(E)** ADE of each serum was evaluated by RT-qPCR measuring the relative viral RNA load in BMDMs when serum was pre-incubated with the DENV (MOI = 0.1). Data are represented as 2^−△△CT^. △CT_sample_ = CT_DENV_ − CT_actin_, △△CT_sample_ = △CT_sample_ − △CT_mork_. Data are the mean ± SEM. Experiments were conducted independently in triplicate. In 2D and 2E, adjusted p-values were calculated by one-way ANOVA with Tukey’s multiple-comparisons test. *ns*: *p* > 0.05, *p < 0.05, **p < 0.01, ***p < 0.001, ****p < 0.0001.

To evaluate the immunogenicity of the E13-HPF vaccine, BALB/c mice from the vaccine group were subcutaneously immunized with the E13-HPF vaccine (10 μg) formulated with an equal volume of alum adjuvant. The control group was given the same dose of HPF nanoparticles. All groups were given a booster dose at week-4 and sera were collected at week-6 (**Figure 2A**). **Figure 2B** indicates a significant difference in the serum IgG titer specifically against E13 between the vaccine group and HPF group. The titers of specific IgG against E13 of the four serotypes were ≥10^4^ until week-10 (**Figure S2A**). Hence, the E13-HPF vaccine could induce balanced and strong immunogenicity among all four serotypes of the DENV. To ascertain if DENV infection could be blocked by the specific IgG, the nAb level was measured. FRNT showed that the NT50 of four-serotype DENV was in the range 10^2^ to 10^3^ (**Figures 2C**, **S2B**). These data indicated that sera from the vaccine group protected Vero cells or BHK21 cells from DENV infection effectively, although NT50 of DENV-1 and DENV-4 was slightly lower than that of the other serotypes of the DENV.

Except for pre-existing nAbs, rapidly reactivated memory B cells (MBCs) are important for antibody generation in the antiviral immune response (30, 31). Therefore, we isolated lymphocytes from the spleen of each immunized mouse at week-6. The percentages of total IgG1^+^ MBCs (CD19^+^ B220^+^ CD38^+^) and IgG2a^+^ MBCs were determined by flow cytometry (**Figure S2C**). Vaccine-immunized mice induced more IgG1^+^ MBCs and IgG2a^+^ MBCs (**Figure 2D**) than those of the control group.

To determine the ADE induced by vaccines *in vitro*, we applied a detection system based on bone-marrow-derived macrophages (BMDMs) from BALB/c mice. Studies have shown that ADE is an obstacle to developing a DENV vaccine because non-nAb or sub-nAb concentrations of DENV-specific antibodies can enhance viral replication in phagocytes expressing Fc receptors (13, 32, 33). In our study, diluted serum was pre-incubated with the DENV to form immune complexes and then the mixture was added to BMDMs. Cells were harvested after 24 h and RNA viral loads were detected by qRT-PCR. At a final serum dilution of 1:50, when different serotypes of the DENV infected BMDMs, the relative viral RNA load in BMDMs presented different fold increases with DENV-2-infected serum from wild-type mice (**Figure 2E**). However, significant enhancement of the relative viral RNA load in BMDMs in the presence of mice serum from the vaccine group was not observed. Hence, the E13-HPF vaccine that enriched E13 antigens in ferritin nanoparticles evoked a robust humoral immune response against the four serotypes of the DENV without the risk of ADE.

### 3.3 E13-HPF vaccine induces strong T-cell immune responses

**Figure 3:**
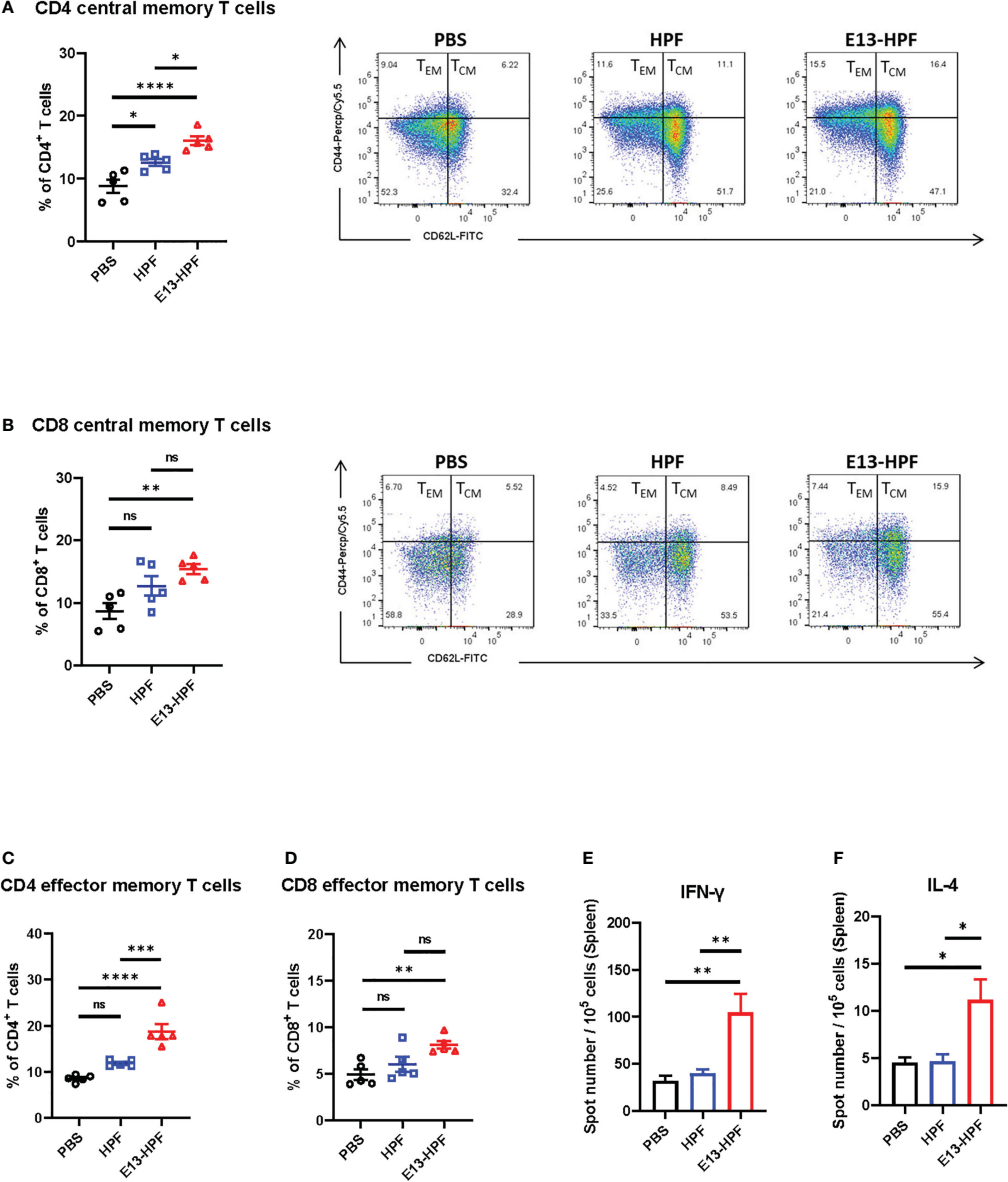
E13-HPF vaccine immunization induces strong cellular immune responses in BALB/c mice. The spleen from each mouse was collected to detect the T-cell phenotypes by flow cytometry at week-6 (n=5). **(A, B)** Percentages of total CD4^+^ central memory T cells (CD4^+^CD62L^+^CD44^+^) and total CD8^+^ central memory T cells (CD8^+^CD62L^+^CD44^+^). **(C, D)** Percentages of total CD4^+^ effector memory T cells (CD4^+^CD62L^-^CD44^+^) and total CD8^+^ effector memory T cells (CD8^+^CD62L^-^CD44^+^). **(E, F)** ELISpot assays were conducted for IFN-γ and IL-4 secretion in splenocytes at week-26 (n=5). Splenocytes were stimulated with DENV-2 DIII. Data are the mean ± SEM. Adjusted p-values were calculated by one-way ANOVA with Tukey’s multiple-comparisons test. *ns*: *p* > 0.05, *p < 0.05, **p < 0.01, ***p < 0.001, ****p < 0.0001.

To evaluate the T-cell immune responses induced by the E13-HPF vaccine, we investigated central memory T (T_CM_) cells and effector memory T (T_EM_) cells in the spleen of mice 2 weeks after boost vaccination. Although clear understanding of cellular immunity in preventing DENV infection is lacking, studies have shown that antiviral CD8^+^ and CD4^+^ T cells play a key part in this process (34, 35). Recent studies have revealed that CD4^+^ T_EM_ cells and CD8^+^ T_EM_ cells function efficiently upon DENV challenge (35, 36). Exposure to pathogens can aid the corresponding self-renewal and differentiation of T_CM_ cells into T_EM_ cells to support further protection. We used flow cytometry to monitor these processes. The gating strategies of flow cytometry are shown in **Figure S2D**. Populations of CD4^+^ T cells and CD8^+^ T cells were defined further as T_CM_ cells (CD62L^+^ CD44^+^) and T_EM_ cells (CD62L^-^ CD44^+^). The E13-HPF vaccine increased the numbers of total CD4^+^ T_CM_ cells and CD8^+^ T_CM_ cells in the spleen significantly (**Figures 3A, B**). A higher percentage of CD4^+^ T_EM_ cells and CD8^+^ T_EM_ cells were activated in the nanoparticle-vaccinated group (**Figures 3C, D**) than in the control group, which indicated that the E13-HPF vaccine induced strong and long-term immune memory. Further, we detected DENV-specific T cells responses using DENV-2 DIII as a stimulus by ELISpot assay. Compared with other groups, we found that the vaccine group elicited more DIII-specific IFN-γ-secreting (Th1-biased cells) and IL-4-secreting (Th2-biased cells) splenocytes (**Figures 3E, F**). In the vaccine group, the number of IFN-γ-secreting splenocytes was much higher than that of IL-4-secreting splenocytes, indicating that E13-HPF vaccination drived Th1-biased cell responses in BALB/c mice.

### 3.4 E13-HPF vaccine protects AG6 mice from DENV challenge

**Figure 4:**
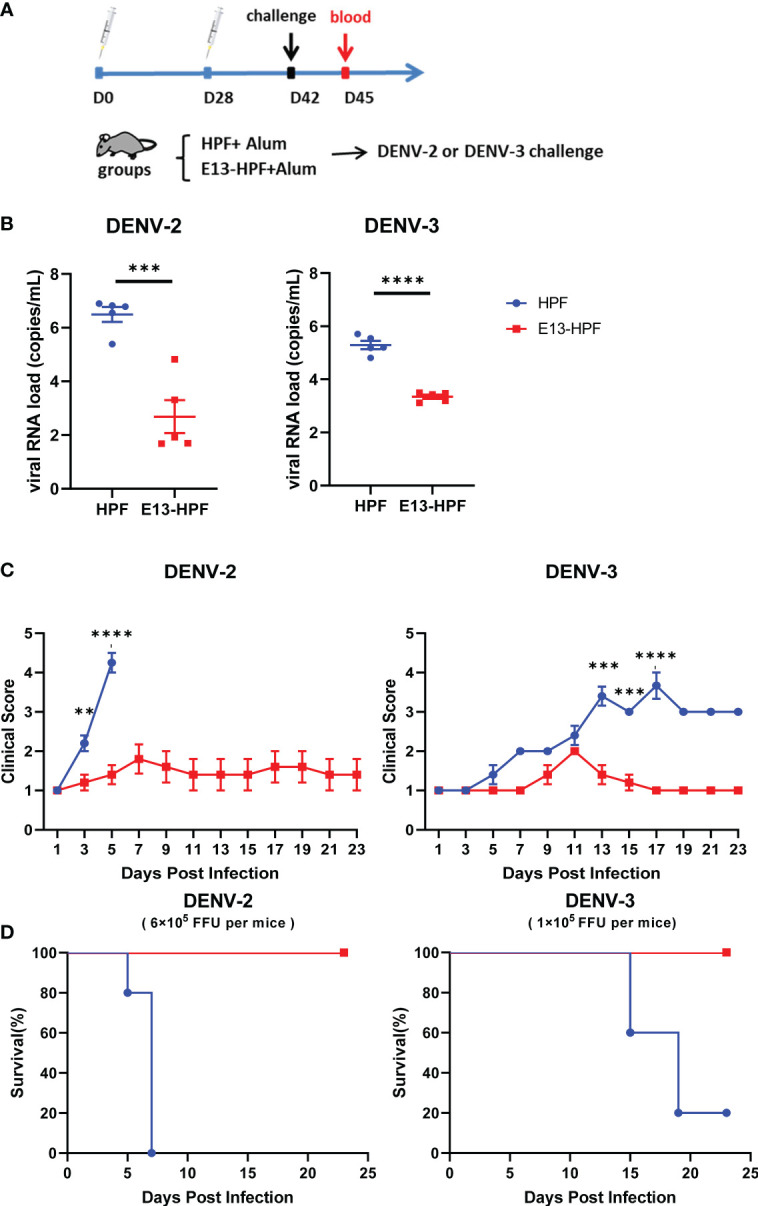
E13-HPF vaccine protects AG6 mice from DENV-2 and DENV-3. **(A)** Vaccination schedule for AG6 mice (schematic). Five mice within each group were prime/boost-vaccinated with tetravalent E13-HPF nanoparticle vaccine on days 0 and 28. Two weeks post-boost, mice were intravenously injected with DENV-2 (6×10^5^ FFU) and DENV-3 (1×10^5^ FFU). **(B)** Detection of viremia at 3 days post-infection. RT-qPCR was executed to measure the viral RNA load in serum. Data were analyzed by the Student’s *t*-test. **(C)** Clinical score of each challenged mouse during 23 days. Score scale is 1 to 5 where: 1 = “healthy”; 2 = “mild signs of lethargy”; 3 = “lethargy, ruffled fur, and hunched posture”; 4 = “lethargy, ruffled fur, hunched posture, and reduced mobility”; 5 = “moribund”. Mice were euthanized if the clinical score was up to 5. Clinical scores were compared every 2 days by the Student’s *t*-test with Holm–Sidak correction. **(D)** Kaplan–Meier survival curves of mice infected with DENV-2 or DENV-3. Data are the mean ± SEM. *ns*: *p* > 0.05, **p < 0.01, ***p < 0.001, ****p < 0.0001.

To further investigate the protective efficiency of the E13-HPF vaccine, the same immune strategy was conducted in AG6 mice (**Figure 4A**). AG6 mice (which are deficient in IFN-α/β and IFN-γ receptors) have been reported to be susceptible to DENV infection and are used frequently in assessment of DENV vaccines (37, 38). Two weeks after the boost, two groups of vaccinated mice were challenged separately with a lethal intravenous dose of DENV-2 (16881 strain) and DENV-3 (isolated clinical strain D191267). Viral RNAs in mice sera were detected by qRT-PCR at 3 dpi. The mean viral RNA load in sera reached 10^6^ copies/mL and 10^5^ copies/mL in the groups of HPF-immunized mice infected with DENV-2 and DENV-3, respectively (**Figure 4B**). In contrast, for most mice vaccinated with tetravalent E13-HPF nanoparticle, the viral RNA load was below the limit of detection. In addition, the clinical score of each mouse was recorded every 2 days. If mice exhibited overwhelming signs of severe disease, they were euthanized. DENV-2-infected mice in the control group started to show lethargy, ruffled fur, and a hunched posture on day-3, and the clinical score of each mouse was ≥4 on day-5 (**Table S1**). The mice in the vaccine group had a clinical score ≤3, and all the mice survived at the end of the experiment (**Figures 4C, D**). Compared with mice challenged with DENV-2, the signs of severe disease in DENV-3-infected mice appeared later. Two mice in HPF-immunized group displayed reduced mobility on day-13 and the number of survival mice declined on day-15. However, mice in the vaccine group were essentially healthy. Taken together, these results demonstrated that the E13-HPF vaccine provided AG6 mice with full protection against DENV-2 and DENV-3.

### 3.5 Single-dose, prime-boost, and third-dose strategies of E13-HPF vaccine exhibited potent protection in BALB/c mice

**Figure 5:**
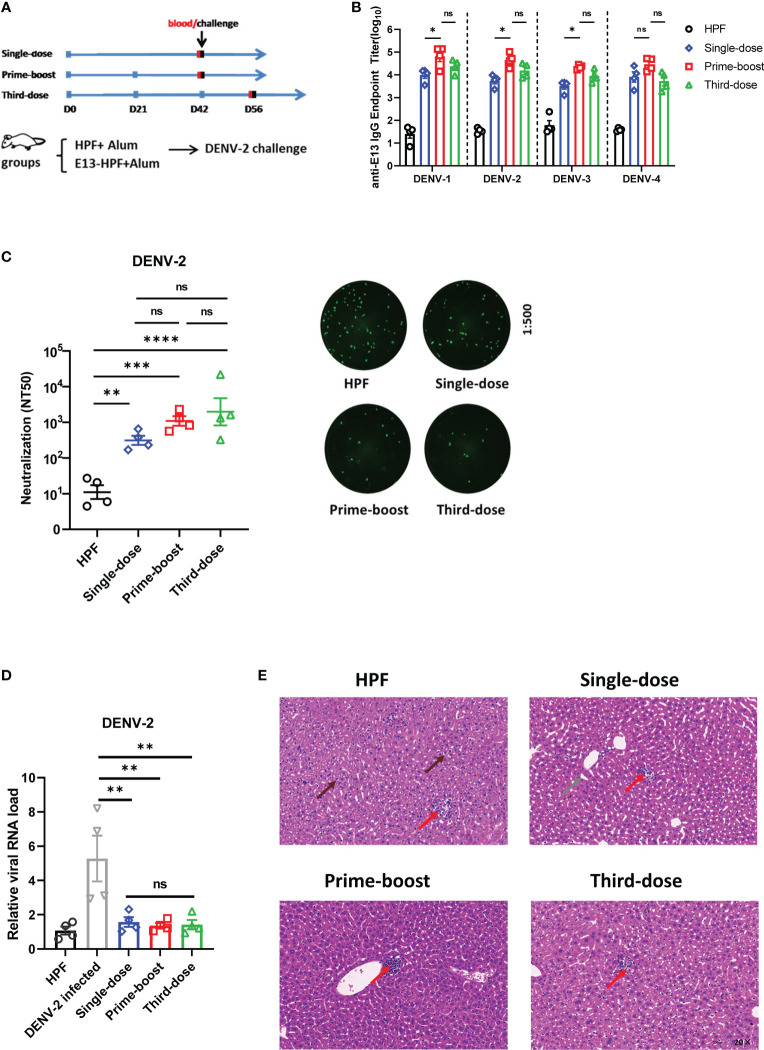
Exploration of different-dose E13-HPF vaccine immunization in BALB/c mice. **(A)** Schedule of different immune strategies (schematic). For the single-dose strategy, mice were immunized on day-0 and challenged with DENV-2 on day-42. For prime-boost and third-dose strategies, mice were vaccinated every 3 weeks. Mice in single-dose and prime-boost groups were challenged on day-42, and those in third-dose group on day-56. All mice were bled on the day before challenge and were euthanized 7 days post-infection. **(B)** Detection of anti-E13 IgG titer before challenge. HPF-immunized mice were used as control. Data were analyzed by two-way ANOVA with Turkey’s multiple-comparisons test. **(C)** Serum titers of neutralizing antibody against DENV-2. Data are represented by NT50 (left). The representatives of FRNT fluorescent spot wells (right) within groups with a final dilution of 1:500. **(D)** ADE evaluation of each group in BMDMs. **(E)** Histopathology of the liver of BALB/c mice. Liver degeneration and eosinophilic granules (brown arrow), widening of sinusoidal spaces (gray arrow), and lymphocytes (red arrow). Data are the mean ± SEM. The data of 5C and 5D were analyzed by one-way ANOVA with Turkey’s multiple-comparisons test. ns: p > 0.05, *p < 0.05, **p < 0.01, ***p < 0.001, ****p < 0.0001.

To determine the protective ability with different doses of the E13-HPF vaccine, single-dose, prime-boost, and third-dose immunization strategies were employed in BALB/c mice (**Figure 5A**). The immune interval was 3 weeks. For single-dose and prime-boost strategies, mouse sera were harvested to measure the titer of specific IgG and nAbs on day-42, whereas sera in the third-dose group were obtained 2 weeks after the final dose. All mice generated a high level of four serotypes of E13-specific IgG at approximate titers of 10^4^, but the prime-boost and third-dose strategies evoked a higher titer than the single-dose strategy (**Figure 5B**). Moreover, anti-DENV-2 nAb titers seemed to be increased in groups with a boost or booster dose, though none of these differences were significant (**Figure 5C**). Significant differences in IgG titers and nAb titers were not found between prime-boost and third-dose groups. ADE assays with BMDMs were also carried out to confirm if different immune strategies influenced ADE. qRT-PCR data revealed that the sera in vaccine groups did not increase the relative viral RNA load (DENV-2) in BMDMs, whereas sera from the DENV-2-infected group resulted in a 5-fold increase (**Figure 5D**). Studies have illustrated that immunocompetent mice (such as BALB/c mice) showed impairment of liver function when they underwent intravenous inoculation of a high dose of an adapted DENV-2 strain (39, 40). Therefore, immunized BALB/c mice were injected (i.v.) with 10^7^ FFU of the 16881 strain of DENV-2 on the day after blood collection. Histopathology of the liver in HPF-immunized mice revealed infiltration of inflammatory cells, signs of necrosis, and cytopathic effects (e.g., intracellular vacuole formation and particulate degeneration) at 7 dpi (**Figures 5E**, **S3**). In contrast, H&E staining of the liver from the single-dose group showed normal hepatocytes even though there was slight widening of sinusoidal spaces. For prime-boost and third-dose groups, almost no impairment was seen in liver sections. Taken together, these results indicated that different dosing strategies of the E13-HPF vaccine protected mice from DENV-2 infection efficiently.

### 3.6 Adjuvant treatment with c-di-GMP improves the efficiency of E13-HPF vaccine in BALB/c mice

**Figure 6:**
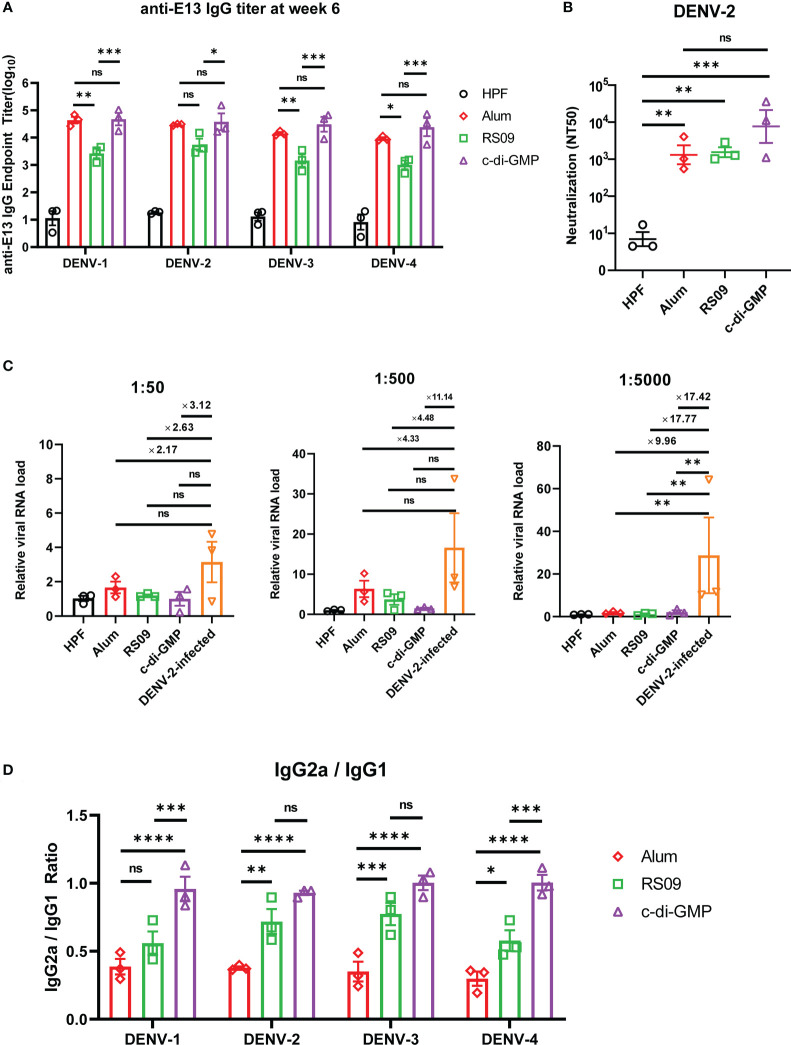
Evaluation of E13-HPF vaccine immunization with different adjuvants in BALB/c mice. Three mice in each group were immunized (s.c.) with 10 µg of the tetravalent E13-HPF nanoparticle vaccine with different adjuvants at weeks 0 and 4. Blood collection was executed at week-6. HPF nanoparticles with alum as control. **(A)** ELISA detection of anti-E13 IgG. **(B)** Serum titers of neutralizing antibodies against DENV-2 are represented by NT50. **(C)** ADE evaluation of each group in BMDMs. DENV-2 (MOI = 0.1) was mixed with serum at a final dilution of 1:50, 1:500 and 1:5000. **(D)** Anti-E13 IgG2a/IgG1 ratio. IgG1 and IgG2a titers were determined by ELISA. Data were represented by the reciprocal of the endpoint serum dilution, and then the IgG2a/IgG1 ratio was calculated. Data are the mean ± SEM. The data of 6A and 6D were analyzed two-way ANOVA with Turkey’s multiple-comparisons test. The data of 6B and 6C were analyzed by one-way ANOVA with Turkey’s multiple-comparisons test. *ns*: *p* > 0.05, *p < 0.05, **p < 0.01, ***p < 0.001, ****p < 0.0001.

To investigate which adjuvants could facilitate E13-HPF vaccine immunogenicity, we compared immune responses in vaccinated BALB/c mice with several types of adjuvants: four TLR agonists (Pam2CSK4, Poly I: C, Vesatolimod, RS09), a cyclic dinucleotide (c-di-GMP), and alum (**Table S3**). Among these adjuvants, RS09 and c-di-GMP could induce a high nAb titer (**Figure S4**). We found that c-di-GMP could induce a higher titer of E13-specific IgG (**Figure 6A**). The nAb titer in the c-di-GMP-treated group reached 10^4^ in two of three samples, which was 10-times higher than that in the alum-treated group, whereas RS09 stimulated the same level of nAbs as alum (**Figure 6B**). To further evaluate ADE under different levels of antibodies, mouse sera from different groups were diluted at 1:50, 1:500, and 1:5000. The relative viral RNA load in BMDMs was enhanced in the DENV-2-infected group with an increase of sera dilution, whereas obvious enhancement was not seen in adjuvant groups (**Figure 6C**). At a sera dilution of 1:5000, the relative viral RNA load in the c-di-GMP group was 17.42-times lower than that in the DENV-2 infected group. At a sera dilution of 1:500, the relative viral RNA load in the c-di-GMP group was 11.14-times lower than that in the DENV-2-infected group, though a significant difference was not observed among groups. These data indicated that the c-di-GMP group showed a stronger ability to avoid ADE than the alum group.

A high IgG2a/IgG1 ratio has a close correlation with the Th1 cell-biased immune response, which enhances protection against virus infection (41). Hence, we examined whether c-di-GMP could induce a stronger Th1-biased immune response. ELISA revealed that RS09 and c-di-GMP stimulated higher E13-specific IgG2a titers than that stimulated by alum (**Figure 6D**). These results indicated that c-di-GMP evoked the highest level of IgG2a, though there were no significant differences in the level of total IgG between alum- and c-di-GMP-treated groups. Taken together, these data implied that c-di-GMP was a more promising adjuvant for the E13-HPF vaccine.

## 4 Discussion

DENV infection is a serious public-health problem in tropical and subtropical regions. Hence, development of a reliable vaccine against DF is necessary. The E protein of the DENV is essential for interactions with multiple receptors for the DENV, including heparin sulfate, laminin LAMR1, and dendritic cell-specific intercellular adhesion molecule-3-grabbing non-integrin (DC-SIGN), during the adhesion and entry of the DENV (42, 43). Isolated antibodies targeting complex epitopes (e.g., DI/DIII region) have highly potent protective activity against the Zika virus and DENV (44, 45). Hence, the DI/DIII region has become a promising target for the development of DENV vaccines. In our study, the modified E protein (containing DI and DIII domains) retained more neutralizing epitopes compared with that in the DIII domain only. More importantly, this design could help to avoid the risk of ADE because the ADE-associated domain (DII) in E protein were removed (33).

Strong immune responses were evoked in BALB/c mice by displaying E13 protein on our ferritin-nanoparticle platform. These data revealed that the E13-HPF vaccine stimulated similar high-level IgG titers against E13 proteins of the four serotypes. However, there are different genotypes within each DENV serotype. Studies have shown that different genotypes may modulate vaccine efficacy, which could lead to a breakthrough infection of viruses of identical serotype (46, 47). In the present study, the DENV strains used in FRNT were different from vaccine antigen strains except for DENV-2. It is possible that some genotypic variation in the DIII domain resulted in relatively poor neutralizing abilities against DENV-1 and DENV-4 (**Figure S1D**). Nevertheless, the average NT50 of vaccine-elicited specific nAbs (against DENV-1, DENV-3, or DENV-4) remained >10^2^, which indicated that the E13-HPF vaccine induced potent production of nAbs. Compared with the control group, there were more IgG1^+^ MBCs and IgG2a^+^ MBCs in the vaccine group (especially IgG2a^+^ MBCs), which indicated that the E13-HPF vaccine could induce durable humoral immune responses. Moreover, T cells can increase antibody responses through coordination between T cells and B cells (48). A high percentage of T_CM_ cells and T_EM_ cells in vaccinated mice (CD4^+^ cells or CD8^+^ cells) suggested that strong cellular immune responses probably aided the prevention of severe diseases. More IFN-γ-secreting T cells (Th1-biased cells) provided safer T cell immune responses. As such, the strong immune responses induced by the E13-HPF vaccine are based on a combination of humoral and cellular immunity.

ADE assessment is vital for the development of a dengue vaccine. Cell lines expressing human Fcγ receptors [e.g., K562 (49, 50)] are often used to evaluate the DENV infection-enhancing activity of mice or human antibodies, but the results are sometimes inconsistent with *in vivo* conditions (15). We used BMDMs to analyze ADE, which is a more reliable method for the detection of ADE *in vitro* (51, 52). The absence of ADE even at low vaccine-elicited antibody concentrations verified our immunogen design and might also have benefited from the balanced and strong immune responses evoked by enriched antigen presentation *via* a ferritin-based nanoparticle platform.

Immune doses and adjuvants have important roles in immunization. In our investigation of immune doses, we discovered that even a single dose of our E13-HPF vaccine could inhibit virus infection efficiently without ADE and reduce the liver impairment caused by challenge with high-dose DENV-2 in BALB/c mice. Usually, third-dose strategies are used in research for DENV vaccines. However, the prime-boost strategy was sufficient for protection against DENV infection because there was no difference between the prime-boost group and third-dose group, which makes the E13-HPF vaccine more economical.

Apart from antigen design, the adjuvant is another way to improve the protective efficiency of a DENV vaccine and prevent ADE (53). In our study, c-di-GMP showed advantages in improving vaccine protection and reducing ADE risk because it elicited a stronger Th1-biased immune response. c-di-GMP is a stimulator of interferon genes (STING) protein that is a potent inducer of type-I interferons (IFNs) (54). IFNs can activate immune cells and disturb virus replication effectively. Moreover, c-di-GMP is likely to induce robust CD8^+^ T-cell responses *via* the STING pathway, which is vital for viral vaccines (53). A recent study stated that a lower dose of nanoparticulate c-di-GMP could lead to expansion of the number of follicular helper T cells and promotion of germinal-center induction (55). For the convenience of vaccine production and enhancement of vaccine efficiency, c-di-GMP could be considered an effective adjuvant delivered by our ferritin-nanoparticle platform.

In our study, all immunized mice were given a dose of 10 μg (i.e., only 2.5 μg per serotype immunogen) and they did not show any side-effects throughout the study. Although more detailed parameters of our vaccine must be assessed further (including long-term immunity monitoring and ADE assessment *in vivo*), all our results indicate that the E13-HPF vaccine is a promising candidate as a DENV vaccine owing to its robust protective efficiency, absence of ADE, and easy production. In addition, the high yield and purity of E13 proteins of four serotypes, as well as their high conjugation efficiency with ferritin, could benefit industrial development.

## Data availability statement

The original contributions presented in the study are included in the article/**Supplementary Material**. Further inquiries can be directed to the corresponding authors.

## Ethics statement

The animal study was reviewed and approved by Zhongshan School of Medicine of Sun Yat-sen University on Laboratory Animal Care.

## Author contributions

QC designed the project and carried out the experiments. RC, HZ, and RL provided valuable guidance for research. RL, BW, and XZ assisted carrying out experiments. QC, RC, and HZ analyzed the data. QC, RC, and HZ wrote the manuscript. All authors contributed to manuscript-writing and approved the submitted version.
