# Supplementary material for: A tetravalent nanoparticle vaccine elicits a balanced and potent immune response against dengue viruses without inducing antibody-dependent enhancement
Source: Front Immunol. 2023 May 19;14:1193175. doi: 10.3389/fimmu.2023.1193175 (PMC10235449; doi:10.3389/fimmu.2023.1193175)
Supplement: Supplementary file 1 [file DataSheet_1.pdf]

Supplementary Figures

Figure S1

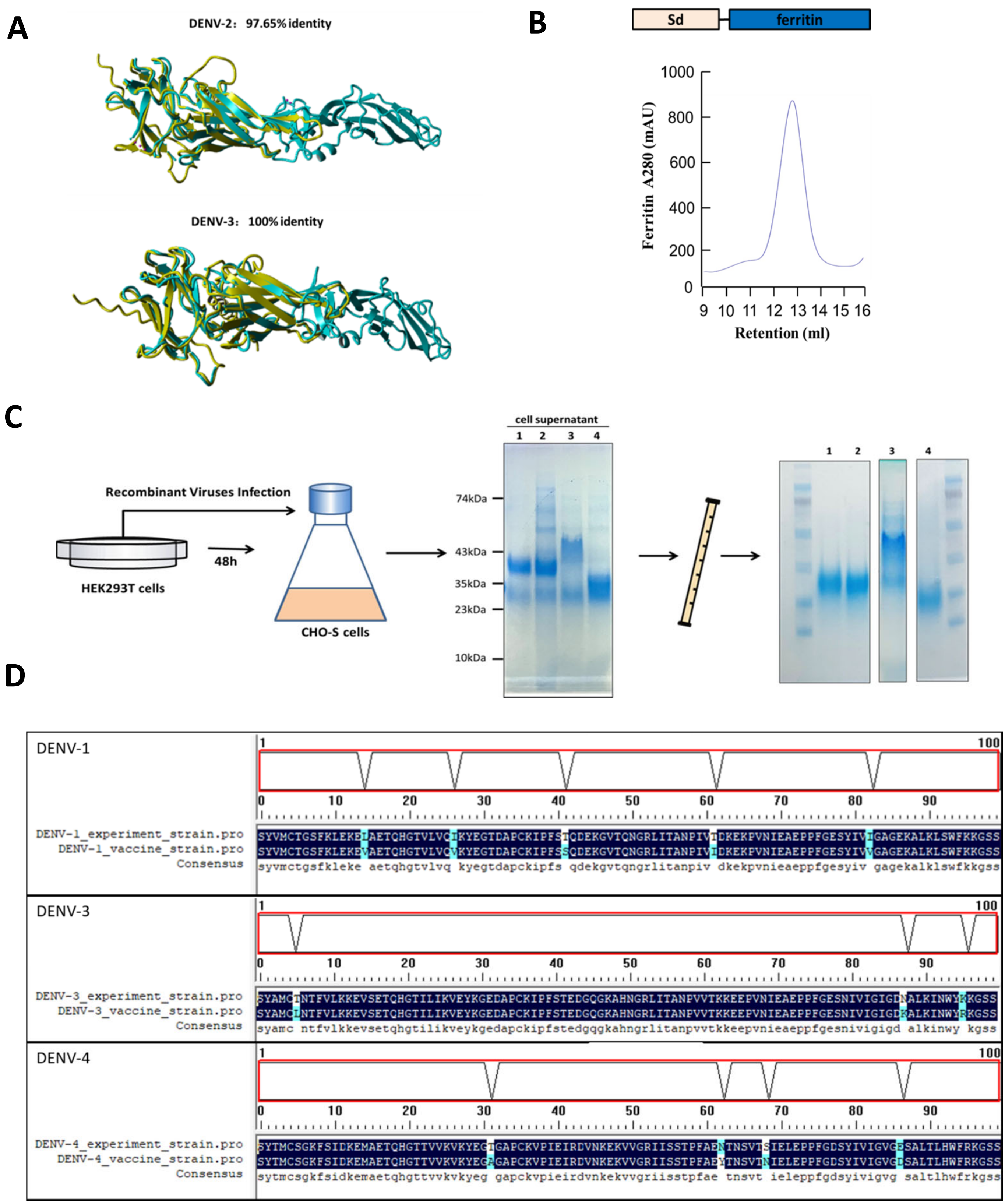

Figure S1. Construction of the E13-HPF vaccine

(A) The structural alignment between E13-Gv predicted by Alpha and natural E protein using MUSTANG *via* YASARA (56). Structure of natural E protein from DENV-2 (UniProtKB: P29990) and DENV-3 (UniProtKB: P27915) were provided in the website of UniProt. Yellow: E13-Gv, Blue: natural E protein. (B) Scheme and SEC of Sd-ferritin. Sd, SdCatcher. (C) Expression and purification of E13-Gv. Coomassie blue staining were conducted to confirm four expressed E13-Gv in cell supernatant and purified protein via SEC. More details in progress were described in method. Lane 1: E113-Gv (DENV-1), lane 2: E213-Gv (DENV-2), lane 3: E313-Gv (DENV-3), lane 4: E413-Gv (DENV-4). (D) Amino acid sequence blasting of vaccine strains and experiment strains. The results were provided by DNAMAN.

Figure S2

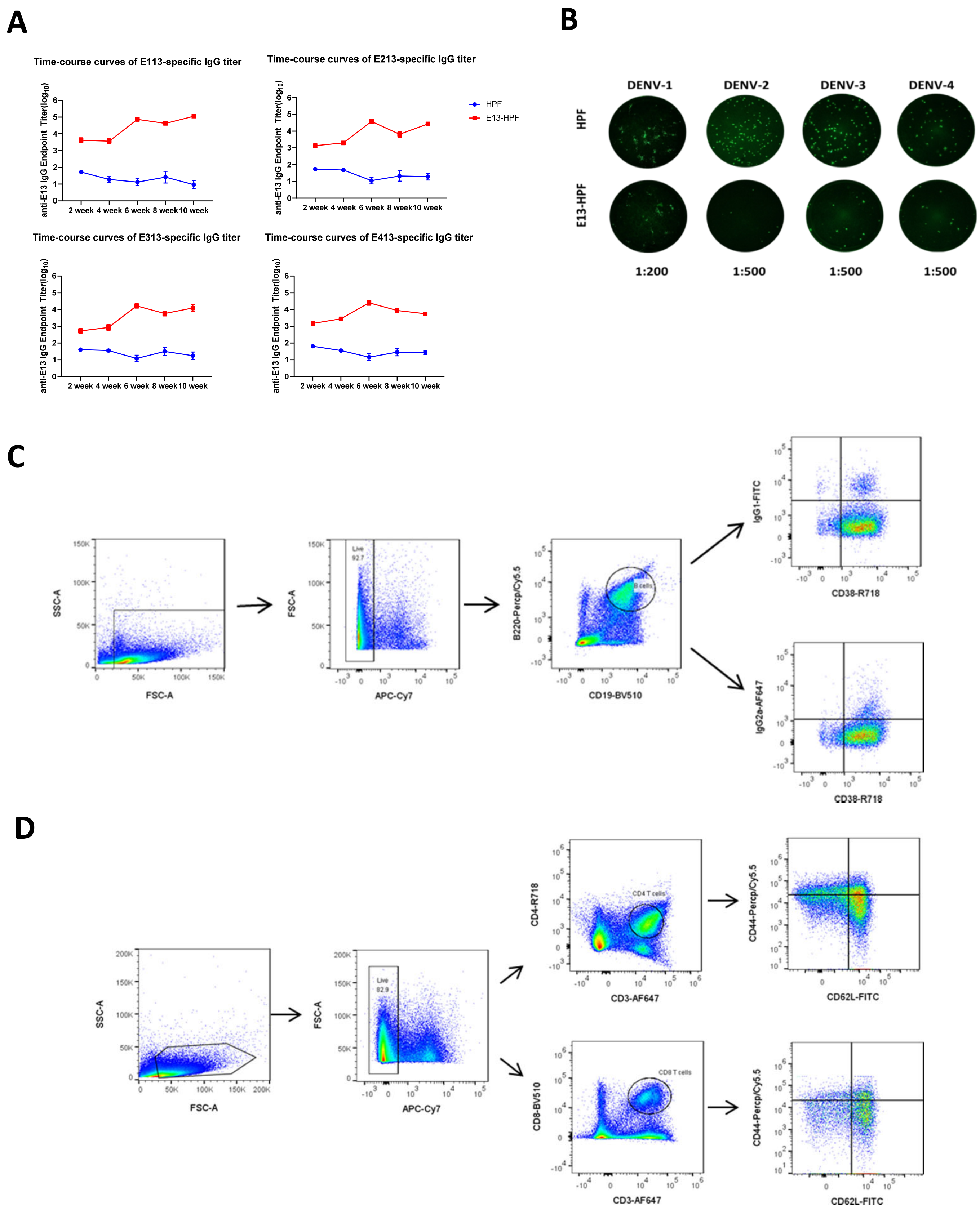

Figure S2. E13-HPF vaccine induces potent humoral immunity in mice

Five mice in each group were immunized at week 0 and week 4. (A) ELISA detection of specific IgG at week 2, 4, 6 and 10. Data were represented as mean ± SEM. (B) Detection of serum nAbs in immunized BALB/c mice. The representatives of FRNT fluorescent spot wells were shown with a final serum dilution of 1:500 at week 6. (C) Gating strategy to analyze the percentages of total IgG1^{+} and IgG2a^{+} MBCs (CD19^{+} B220^{+} CD38^{+}) in the spleen by flow cytometry. (D) Gating strategy to define T cell subsets after staining against CD3, CD4, CD8, CD44 and CD62L.

Figure S3

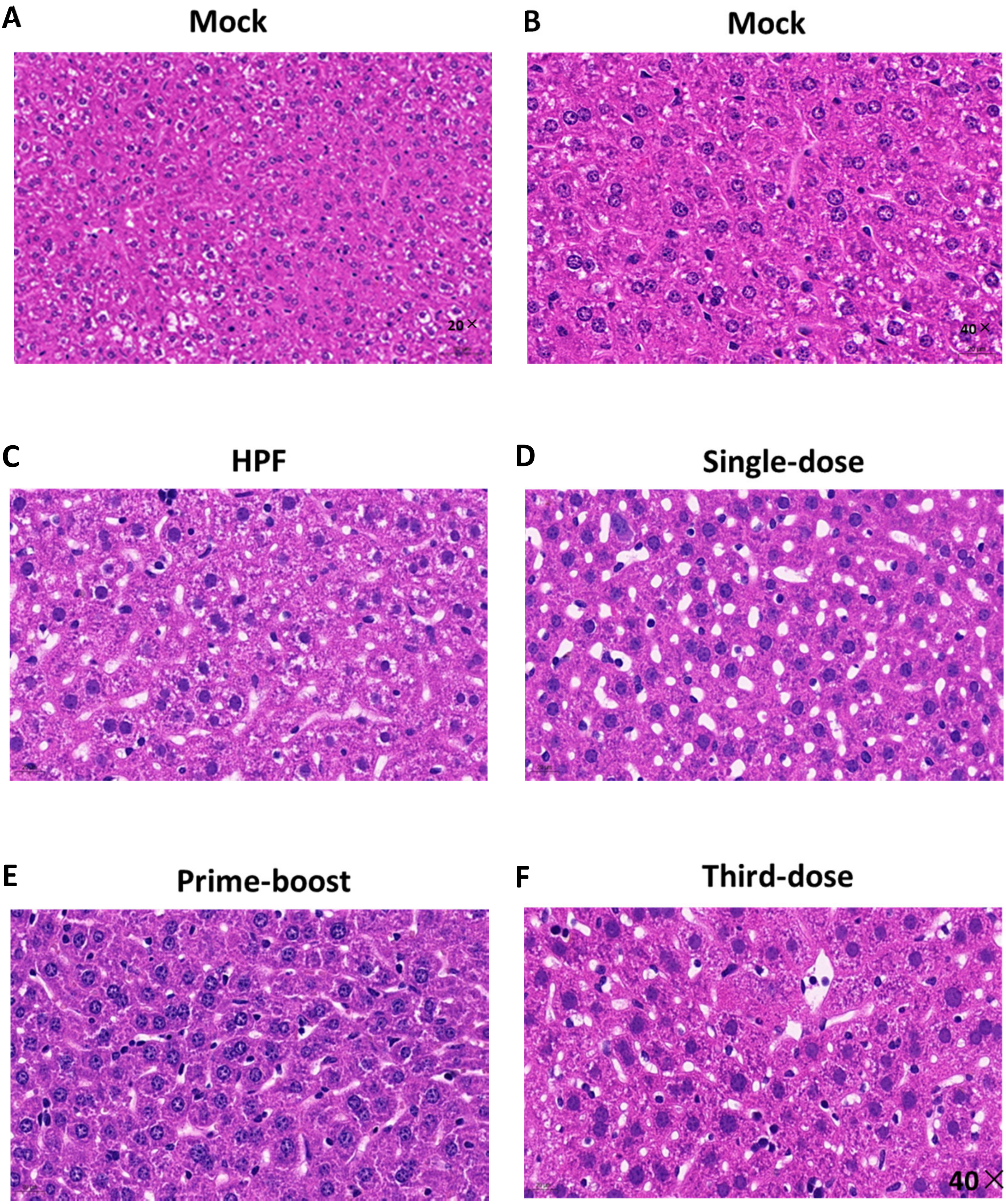

Figure S3. Histopathological analysis of DENV-2 infected BALB/c in different groups
(A-B) H&E staining from mock group in a 20× and 50× field of view. (C) Abundant hepatocytes degeneration in HPF group. (D-F) Round and undamaged liver cells in vaccinated groups.

Figure S4

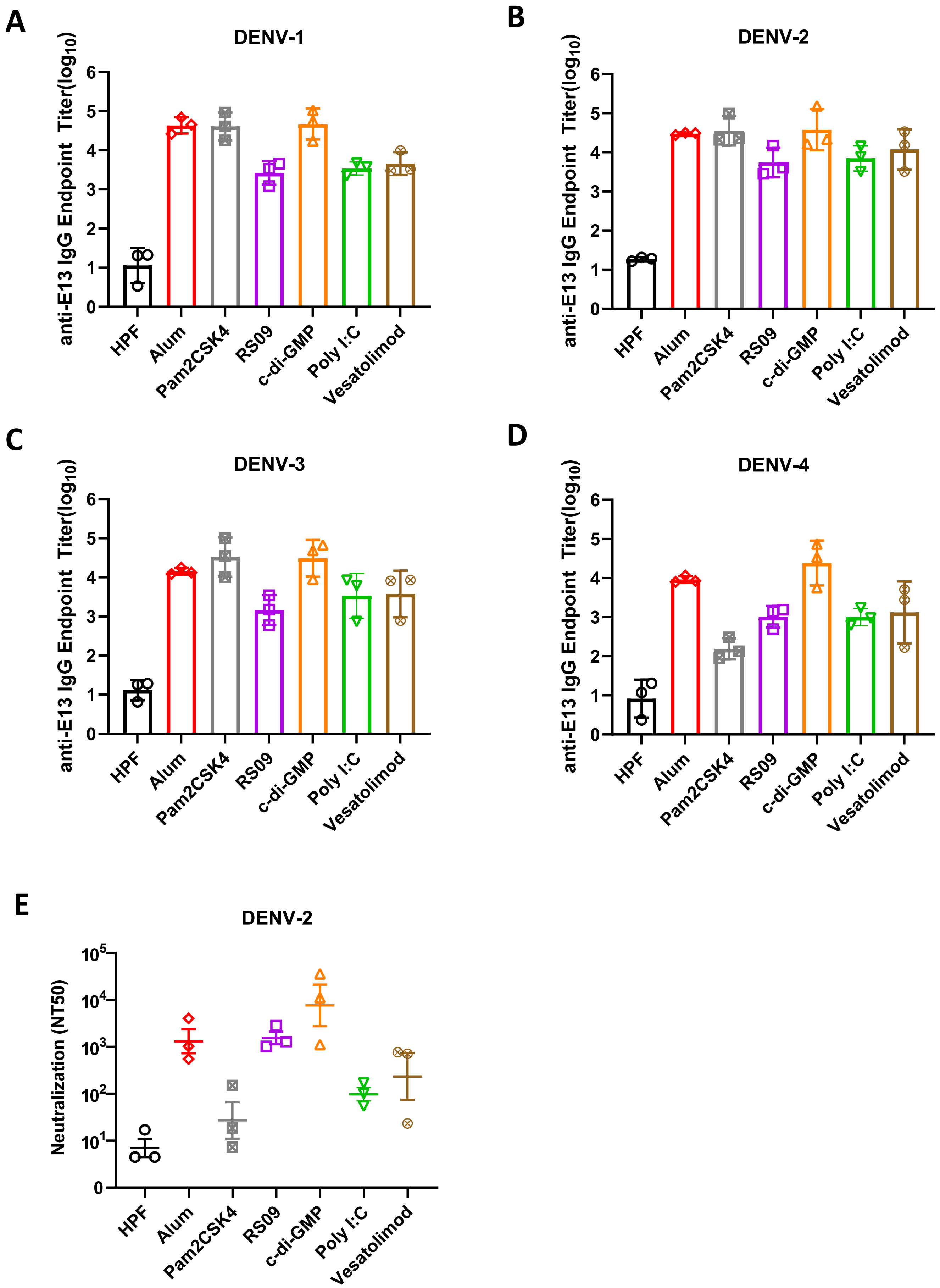

Figure S4. Immunogenicity analyses of vaccinated BALB/c mice in different adjuvant groups

(A-D) ELISA detection of different serotypes of anti-E13 IgG titer. (E) Detection of serum neutralizing antibody titers against DENV-2.
