# Supplementary material for: A tetravalent nanoparticle vaccine elicits a balanced and potent immune response against dengue viruses without inducing antibody-dependent enhancement
Source: Front Immunol. 2023 May 19;14:1193175. doi: 10.3389/fimmu.2023.1193175 (PMC10235449; doi:10.3389/fimmu.2023.1193175)
Supplement: Supplementary file 2 [file DataSheet_2.pdf]

Reference in Supplementary Material

56. Konagurthu AS, Whisstock JC, Stuckey PJ, Lesk AM. MUSTANG*:* a multiple structural alignment algorithm*. Proteins*, (2006) **64**(3): 559-74. doi: 10.1002/prot.20921
