# Supplementary material for: A tetravalent nanoparticle vaccine elicits a balanced and potent immune response against dengue viruses without inducing antibody-dependent enhancement
Source: Front Immunol. 2023 May 19;14:1193175. doi: 10.3389/fimmu.2023.1193175 (PMC10235449; doi:10.3389/fimmu.2023.1193175)
Supplement: Supplementary file 3 [file Table_1.pdf]

Supplementary Tables

Table S1. The recorded clinical score of challenged mice in each group every 2 days

Clinical scoring was based on a scale of 1 to 5: 1, healthy; 2, mild signs of lethargy; 3, lethargy, ruffled fur, and hunched posture; 4, lethargy, ruffled fur, hunched posture, and decreased mobility; 5, moribund. "-" meant that mouse was dead or euthanized.

DENV-2 Infected Mice

Days	HPF					E13-HPF				
1	1	1	1	1	1	1	1	1	1	1
3	2	3	2	2	2	2	1	1	1	1
5	5	-	4	4	4	2	1	1	1	2
7	-	-	-	-	-	3	1	2	1	2
9	-	-	-	-	-	3	1	1	1	2
11	-	-	-	-	-	3	1	1	1	1
13	-	-	-	-	-	3	1	1	1	1
15	-	-	-	-	-	3	1	1	1	1
17	-	-	-	-	-	3	1	1	2	1
19	-	-	-	-	-	3	1	1	2	1
21	-	-	-	-	-	3	1	1	1	1
23	-	-	-	-	-	3	1	1	1	1

DENV-3 Infected Mice

Days	HPF					E13-HPF				
1	1	1	1	1	1	1	1	1	1	1
3	1	1	1	1	1	1	1	1	1	1
5	1	1	2	2	1	1	1	1	1	1
7	2	2	2	2	2	1	1	1	1	1
9	2	2	2	2	2	2	1	1	2	1
11	2	2	3	3	2	2	2	2	2	2
13	3	3	4	4	3	2	1	1	2	1
15	3	3	-	-	3	1	2	1	1	1
17	3	4	-	-	4	1	1	1	1	1
19	3	-	-	-	-	1	1	1	1	1
21	3	-	-	-	-	1	1	1	1	1
23	3	-	-	-	-	1	1	1	1	1

Table S2. Strain-specific primers of DENV detection

Gene	forward primer	reverse primer
DENV-1	5'- GCGAAATCCACTTTCAAGAAATTC-3'	5'- GCCTAGGTCCACGTCTCTTTCATA-3'
DENV-2	5'- TGGGGAAAAGAGAAGACCAATGG-3'	5'- TTCTACCACAGAACTCCTGCTTC-3'
DENV-3	5'- CAGGTGGGAACTTATGGTCTGAA-3'	5'- CCTTTGGTTTCCAGCCATTGTGTA-3'
DENV-4	5'- CTGACAAAACGCAGCAACAAAG-3'	5'- CTGGTCTTTCCCAGCGTCAATAT-3'

Table S3. Different adjuvants in mice immunization

Adjuvant	Targeting	the dosage per mouse
Poly I:C	TLR3	100 μg
Vesatolimod	TLR7	30 μg
Pam_2CSK_4	TLR2/6	7.5 μg
RS09	TLR4	30 μg
c-di-GMP	STING	40 μg
